# Cross-Sectional Associations Between Functional Independence and Behavioral Problems in Children with Cerebral Palsy: The Relational Roles of Family Impact and Maternal Self-Blame

**DOI:** 10.3390/healthcare14142038

**Published:** 2026-07-08

**Authors:** Pınar Algedik, Rahime Gökboğa, Elif İrem Günaydın, Seda Saka, Hatice Gülhan Sözen, Ezgi Şen Yılmaz, Azad Asaf, Selen Kömürcü, Rana Terlemez

**Affiliations:** 1 Department of Psychiatry, Faculty of Medicine, Haliç University, 34060 Istanbul, Turkey; 2 Department of Manager of Barrier-Free Life Application and Research Center, Gedik University, 34060 Istanbul, Turkey; rahime.gokboga@gedik.edu.tr; 3 Physiotherapy Program, Vocational School, Haliç University, 34060 Istanbul, Turkey; iremgunaydin@halic.edu.tr; 4 Department of Physiotherapy and Rehabilitation, Faculty of Health Sciences, Haliç University, 34060 Istanbul, Turkey; sedasaka@halic.edu.tr; 5 Department of Child Neurology, Faculty of Medicine, Bahçeşehir University, 34060 Istanbul, Turkey; drgulhantuncel@hotmail.com; 6 Department of Psychology, Haliç University, 34060 Istanbul, Turkey; drezgisen@gmail.com; 7 Department of Psychiatry, Hitit Üniversitesi, 19040 Corum, Turkey; dr.azadasafov@gmail.com; 8 Psychology, Private Practice, 34060 Istanbul, Turkey; psk.selenkomurcu@gmail.com; 9 Department of Physical Medicine and Rehabilitation, Istanbul University-Cerrahpaşa, 34060 Istanbul, Turkey

**Keywords:** cerebral palsy, CBCL Total Problems, maternal cognitive emotion regulation, maternal self-blame, family impact, parental bonding, parent–child relations

## Abstract

**Highlights:**

**What are the main findings?**

- Children with cerebral palsy exhibit significantly higher behavioral problems across all domains compared to typically developing controls.
- Within the structural model, maternal self-blame is uniquely and independently associated with adverse family impact.

**What are the implications of the main findings?**

- Child behavioral difficulties and family adaptation operate as parallel cross-sectional systems rather than a single interconnected pathway.
- Maternal self-blame serves strictly as a prospective target for future longitudinal research rather than an established therapeutic mechanism.

**Abstract:**

**Background/Objectives**: Children with cerebral palsy, a permanent neurodevelopmental condition that primarily affects movement and posture, exhibit higher rates of behavioral problems. This study examined the cross-sectional associations linking child functional independence in motor and cognitive domains and maternal self-blame to family adaptation and child behavioral presentations within an integrated analytical framework. **Methods**: In a cross-sectional design, 79 child–mother dyads in the cerebral palsy group and 62 dyads in a typically developing control group involving children aged 6 to 18 years were evaluated using the Child Behavior Checklist, Functional Independence Measure for Children, Parental Bonding Instrument, Parent–Child Relationship Scale, Impact on Family Scale, and Cognitive Emotion Regulation Questionnaire. Path analysis using full-information maximum likelihood examined the relational structures exclusively within the cerebral palsy group. **Results**: Group comparisons indicated that the cerebral palsy group scored significantly higher on child behavioral total problems and all syndrome subscales than the controls. Sensitivity analyses revealed that while elevated child behavioral difficulties remained robust after adjusting for demographic imbalances, differences in parental overprotection and maternal self-blame attenuated to non-significance. Within the structural model, maternal self-blame was the only exogenous variable significantly associated with adverse family impact. Direct and indirect relational pathways from child cognitive functional independence and motor parameters to child behavioral total problems through family impact did not reach statistical significance. **Conclusions**: Child behavioral difficulties and family-level adaptation challenges in cerebral palsy represent cross-sectional patterns organized through distinct parallel systems rather than a single interconnected pathway. These findings suggest the potential utility of routine clinical screening for maternal cognitive emotion regulation patterns and identify maternal self-blame as a hypothesis-generating target for future longitudinal intervention trials.

## 1. Introduction

Cerebral palsy (CP) is among the most prevalent neurodevelopmental conditions of childhood, encompassing not only motor impairment but also frequently co-occurring cognitive, behavioral, and emotional difficulties. CP arises from non-progressive disturbances in the developing fetal or infant brain, most commonly attributable to perinatal hypoxia-ischemia, prematurity, stroke, or infection; as such, its etiology is unrelated to parenting behavior or family environment Current systematic analyses estimate the birth prevalence of CP between 1.6 and 3.7 per 1000 live births [1]. In a multicenter study spanning seven European countries and including 818 children with CP, behavioral and emotional problems were identified in approximately one quarter of the sample; the majority of these problems followed a chronic course and placed a considerable strain on family life [2]. Such findings underscore the need for systematic attention to the psychosocial dimensions of CP beyond motor impairment alone.

Population-based evidence consistently shows that behavioral and emotional problems are substantially more common in children with CP than in their typically developing peers. In an Icelandic national cohort of preschool-aged children with CP, 48% scored in the clinical or borderline range on the CBCL Total Problems scale, representing a roughly fourfold increase in risk relative to typically developing peers; attention problems and internalizing symptoms were the most prominent subscales [3]. Intellectual disability and chronic pain have also been identified as independent risk factors for these difficulties [2]. Taken together, these data suggest that behavioral problems in CP cannot be attributed solely to motor limitations and that cognitive and sensory processes also contribute.

Evidence suggests that behavioral problems in children with cerebral palsy are influenced not only by neurological injury, but also by functional independence, participation restrictions, social interaction opportunities, and family/environmental factors operating through complex psychosocial mechanisms. Early influential work by Raina et al. [4] suggested that these relationships may operate both directly and indirectly through family and environmental mechanisms, while more recent evidence has further emphasized the unique contribution of contextual and environmental factors beyond functional limitations [5].

Much of the caregiving burden for children with CP falls on mothers, and several studies have documented that maternal psychological processes can shape the quality of mother–child interaction [6,7]. At the same time, raising a child with CP exerts multilayered effects on the family system. Systematic review evidence identifies the degree of physical disability and caregiver depressive symptoms as primary determinants of caregiving burden [8]. In a structural equation model involving 468 caregivers, Raina et al. [4] found that child behavioral problems were the strongest direct predictor of caregiver psychological health, with family functioning and caregiver self-efficacy also making significant contributions. The relationship between a child’s functional status and behavioral outcomes is therefore not independent of family system dynamics. Mothers of children with CP who exhibit high levels of self-criticism and dependency have been reported to display more pronounced unresolved emotional processes [9], raising the possibility that maternal cognitive emotion regulation capacity may function as an independent factor associated with family dynamics, and potentially with child behavioral outcomes.

Cognitive emotion regulation strategies occupy a central position in shaping responses to stressful life circumstances. Meta-analytic evidence has established that maladaptive strategies such as rumination, avoidance, and suppression are more consistently and strongly associated with psychopathology than adaptive strategies [10]. Self-blame, rumination, and catastrophizing have been identified as stable predictors of depressive symptoms across the lifespan [11]. A further study by the same research group in an adolescent sample showed that self-blame and rumination specifically predicted internalizing problems, whereas externalizing problems were explained by a different mechanism [12]. These findings suggest that cognitive emotion regulation may be closely associated with both the severity and the expression of behavioral problems.

When the existing literature is considered as a whole, child functional limitations in cerebral palsy, family dynamics, and maternal cognitive emotion regulation have largely been examined along separate lines of inquiry. While separate components of these relationships are well-documented, there is a lack of integrated models that simultaneously evaluate how distinct domains of functional independence and specific maternal cognitive emotion regulation strategies relate to family impact and child behavioral presentations. In this framework, the role of maternal cognitive emotion regulation capacity, particularly maternal self-blame, is conceptualized as an independent factor in the sense that its cross-sectional association with perceived family impact is statistically separable from the child’s objective motor and cognitive functional parameters. Whether this independence reflects a stable maternal characteristic or a reaction to unmeasured aspects of the caregiving experience cannot be determined from cross-sectional data and remains an open question for longitudinal research. Furthermore, understanding how these maternal traits are linked with broader variables such as parental bonding and parent–child relations within the family ecology can offer a more nuanced understanding of the systemic factors surrounding child rehabilitation.

To address this gap, the primary aim of this study was to evaluate an observed-variable path model examining the cross-sectional associations among functional independence, maternal self-blame, family impact, and child behavioral problems, quantified as CBCL Total Problems, within a sample of families affected by cerebral palsy. As a secondary aim, given the potential for demographic differences to confound psychosocial outcomes, we sought to compare children with cerebral palsy and a control group of typically developing peers on behavioral problems, parenting variables, and maternal cognitive emotion regulation, while explicitly applying sensitivity analyses to control for demographic imbalances.

## 2. Materials and Methods

### 2.1. Study Design and Setting

This cross-sectional, prospective, single-center, observational-analytic study was conducted between January 2025 and January 2026. Participants were recruited consecutively on a voluntary basis from families attending outpatient and inpatient early intervention and rehabilitation services in Istanbul, Turkey. The target age range for child participants was 6 to 18 years.

### 2.2. Ethical Approval

The study protocol was approved by the Haliç University Non-Interventional Clinical Research Ethics Committee (approval number: 232, date: 27 December 2024). The study was conducted in accordance with the Declaration of Helsinki. Written informed consent was obtained from all participants prior to enrollment; for participants younger than 18 years, legal guardian consent was obtained from the mothers.

### 2.3. Participants and Sample Size Justification

Children aged 6–18 years with a confirmed diagnosis of CP according to the Surveillance of Cerebral Palsy in Europe (SCPE) diagnostic criteria [11], who presented to the outpatient clinic or were admitted for inpatient rehabilitation, along with their mothers, were included in the CP group. Inclusion criteria required a confirmed CP diagnosis and maternal literacy. Children with severe neurodevelopmental or psychiatric conditions, specifically autism spectrum disorder or psychosis, were excluded from the study. Psychiatric evaluations were systematically performed by the child and adolescent psychiatrist on the multidisciplinary team through clinical interviews based on DSM-5 criteria. Children with attention-deficit/hyperactivity disorder (ADHD), anxiety, or depressive disorders were included in the sample. Clinical evaluation of children in the CP group was performed by a multidisciplinary team comprising specialists in physical medicine and rehabilitation, pediatric neurology, and child and adolescent psychiatry. A total of 79 child–mother dyads were enrolled in the CP group through consecutive sampling. Distribution of CP subtypes was recorded according to the SCPE topographic classification [13].

The control group consisted of typically developing children and their mothers who presented for routine check-ups at the well-child unit of the Pediatric Outpatient Clinic at the same institution. Children with any chronic, neurological, or psychiatric condition were excluded. Maternal literacy was also required in the control group. Frequency matching for child age and sex was applied between the CP and control groups. A total of 62 child–mother dyads were enrolled in the control group.

An a priori power analysis was performed for group comparisons to determine the required sample size. Given the moderate-to-large effect sizes (d = 0.50–0.70) reported in comparative studies using the CBCL in children with CP, a conservative approach was adopted with Cohen’s d = 0.55, a two-tailed significance level of alpha = 0.05, and a statistical power of (1 − beta) = 0.80. These parameters indicated a minimum of 53 participants per group; after adding a 15% attrition margin, a target of at least 62 participants per group was set. The study was completed with 79 participants in the CP group and 62 in the control group. The power analysis was conducted using G*Power version 3.1 (Heinrich-Heine-Universität Düsseldorf, Düsseldorf, Germany). For the structural path model, sample size adequacy was initially considered using the conventional benchmark of 10 observations per free parameter. However, because general parameter-to-sample heuristics provide limited guidance for bootstrap-based mediation models involving multiple correlated indirect effects, statistical power for the structural pathways was evaluated using model-specific Monte Carlo simulations. While our analytical sample (*n* = 79) was fully adequate for the medium-to-large effects expected in the primary between-group and bivariate correlational analyses, the Monte Carlo simulation indicated that the final path model was underpowered to detect small-to-medium indirect (mediated) effects (estimated power ≈ 0.35), requiring approximately 225 dyads to reach a target power of 0.80. Consequently, the structural mediation analyses within the cerebral palsy group are treated cautiously as exploratory and hypothesis-generating.

### 2.4. Instruments

Six measurement instruments were administered alongside a sociodemographic information form. The Child Behavior Checklist (CBCL), the Parental Bonding Instrument (PBI), the Parent–Child Relationship Scale (PCRS), and the Cognitive Emotion Regulation Questionnaire (CERQ) were administered to both groups, whereas the Functional Independence Measure for Children (WeeFIM) and the Impact on Family Scale (IFS) were administered only to the CP group. The sociodemographic form collected data on child age, sex, mode of delivery, medical history, educational status, and family socioeconomic characteristics; in the CP group, additional information was obtained on age at CP diagnosis, CP subtype, rehabilitation support, and accompanying medical conditions.

Child Behavior Checklist 6–18 (CBCL/6–18). The Turkish adaptation [14] of the parent-report measure developed by Achenbach and Rescorla [15] to assess children’s behavioral and emotional problems was used. The instrument consists of 120 items scored from 0 (not true) to 2 (very true/often true). Eight syndrome subscales were computed: Anxious/Depressed, Withdrawn/Depressed, Somatic Complaints, Social Problems, Thought Problems, Attention Problems, Rule-Breaking Behavior, and Aggressive Behavior. Three broadband scores were derived: Internalizing (Anxious/Depressed + Withdrawn/Depressed + Somatic Complaints), Externalizing (Rule-Breaking Behavior + Aggressive Behavior), and Total Problems (sum of all 120 items). T-scores below 60 were classified as normal, 60–63 as borderline, and above 63 as clinical [15].

Functional Independence Measure for Children (WeeFIM). The WeeFIM, whose validity and reliability in Turkish children with CP were established by Tur et al. [16], was used to assess the children’s functional independence. The instrument comprises 18 items scored from 1 (total dependence) to 7 (complete independence). Six subscales (Self-Care, Sphincter Control, Transfers, Locomotion, Communication, Social Cognition) are computed, from which Motor (13 items; range 13–91) and Cognitive (5 items; range 5–35) domain scores and a total score (range 18–126) are obtained. Higher scores reflect greater functional independence. The WeeFIM was administered only to the CP group because of ceiling effects in typically developing children.

Parental Bonding Instrument (PBI). The Turkish adaptation by Kapçı and Küçüker [17] of the instrument developed by Parker et al. [18] to measure perceived parental bonding during the first 16 years of life was used. The PBI consists of 25 items scored from 0 (very unlike) to 3 (very like), with 13 items reverse scored. Consistent with the factor structure of the Turkish adaptation, two subscales are computed: Care (18 items; range 0–54) and Overprotection (7 items; range 0–21). Higher scores indicate more favorably perceived parental bonding.

Parent–Child Relationship Scale (PCRS). The Turkish adaptation by Aytaç et al. [19] of the instrument developed by Hetherington and Clingempeel was used to evaluate the quality of the parent–child relationship. Two subscale scores are computed: Positive Relationship (10 items; range 10–50) and Negative Relationship (5 items; range 5–25); no total score is derived.

Impact on Family Scale (IFS). The Turkish adaptation by Beydemir [20] of the instrument originally developed by Stein and Riessman [21] and later psychometrically re-evaluated by Stein and Jessop [22] was used to assess the impact of the child’s condition on the family. The IFS consists of 27 items scored from 1 (strongly agree) to 4 (strongly disagree); three items (items 2, 3, and 5) were excluded from scoring, and the total was computed over 24 items. No items were reverse-scored. Four subscales are calculated: Financial Burden (4 items), Familial/Social Impact (9 items), Personal Strain (6 items), and Mastery (5 items). The Total Impact score is obtained by summing the first three subscales, excluding Mastery (19 items; range 19–76). The Overall Total score is the sum of all 24 scored items (range 24–96). Higher scores indicate greater adverse family impact. The IFS was administered only to the CP group because item content is specific to the context of chronic illness or disability. Following the correction of the scoring-direction error, raw scores were systematically transformed and recoded to ensure that all reported IFS scores were oriented so that higher values indicate greater adverse family impact.

Cognitive Emotion Regulation Questionnaire (CERQ). The Turkish adaptation by Onat and Otrar [23] of the instrument developed by Garnefski et al. [24] was used to assess cognitive coping strategies employed by mothers in response to negative life events. The CERQ consists of 36 items scored from 1 (almost never) to 5 (almost always), with no reverse-scored items. Nine four-item subscales are computed (range 4–20 each): Self-Blame, Acceptance, Rumination, Positive Refocusing, Refocus on Planning, Positive Reappraisal, Putting into Perspective, Catastrophizing, and Blaming Others. These subscales are grouped into two higher-order categories: adaptive strategies (Acceptance, Positive Refocusing, Refocus on Planning, Positive Reappraisal, and Putting into Perspective; range 20–100) and maladaptive strategies (Self-Blame, Rumination, Catastrophizing, and Blaming Others; range 16–80).

### 2.5. Variable Definitions and Endpoints

The dependent variable (outcome) was behavioral and emotional problems as measured by the CBCL. The primary endpoint was the CBCL Total Problems score; secondary endpoints were the CBCL Internalizing and Externalizing scores. Functional independence as assessed by the WeeFIM (Motor and Cognitive domain scores) served as the independent variable. The IFS (family impact) score was designated as a candidate mediator variable, and the CERQ Self-Blame subscale score (cognitive emotion regulation strategy) was designated as the exogenous variable. The PCRS was included as an additional indicator of parent–child relationship quality in group comparisons. Group comparison analyses used the instruments administered to both the CP and control groups (CBCL, PBI, PCRS, CERQ); path analysis models were tested in the CP group only.

### 2.6. Statistical Analysis

Normality of continuous variables was evaluated separately in each group using a multicomponent assessment comprising the Shapiro–Wilk test, skewness and kurtosis coefficients, and visual inspection of histograms, Q–Q plots, and box plots. Normally distributed continuous variables were reported as mean ± standard deviation (SD), and non-normally distributed continuous variables as median [minimum–maximum]. Categorical variables were expressed as frequencies and percentages.

Normally distributed variables—specifically PBI Overprotection and the CERQ Self-Blame, Refocus on Planning, Positive Reappraisal, and Putting into Perspective subscales—were compared using the independent-samples Student’s *t*-test, or Welch’s *t*-test when Levene’s test indicated unequal variances, with Cohen’s d [95% confidence interval (CI)] as the effect size. All remaining continuous variables, which violated the normality assumption, were compared using the Mann–Whitney U test, with the rank-biserial correlation [95% CI] as the effect size; confidence intervals for the rank-biserial correlation were obtained by bias-corrected bootstrapping. Categorical variables were analyzed with Pearson’s chi-square test or the Fisher–Freeman–Halton exact test depending on expected cell frequencies, with Cramer’s V as the effect size. As a sensitivity analysis, because the groups differed on several demographic variables, each CBCL outcome (standardized) was additionally regressed on group, child age, sex, and maternal education using ordinary least squares with heteroscedasticity-consistent (HC3) standard errors. The same covariate-adjusted model was applied to the parenting (PBI, PCRS) and maternal cognitive emotion-regulation (CERQ) outcomes.

Associations among key variables in the CP group were examined using Spearman’s rank correlation coefficient (rho), with 95% confidence intervals, and were additionally examined as partial correlations adjusting for child age, sex, and maternal education. An observed-variable path model was then estimated to examine the indirect relational structures among functional independence (WeeFIM Motor and Cognitive), maternal self-blame, family impact (IFS Total Impact), and child behavioral problems, with family impact specified as a candidate mediator. The model was estimated by full-information maximum likelihood (FIML); standardized coefficients are reported, and indirect effects were evaluated with bootstrap 95% confidence intervals. The Parental Bonding Instrument was not included as a mediator in the structural path model because it was completed by mothers regarding their own parents and therefore reflects the mothers’ own developmental history rather than the current mother–child relationship.

Missing data were confined to the maternal self-report instruments (IFS, 21.5%; CERQ, 22.8%; PBI, 19.0% to 20.3%; PCRS, 21.5%), whereas the clinician-rated WeeFIM and the CBCL had no missing values. Within the CP group, cases with complete versus incomplete path-model data did not differ in child age (*p* = 0.42), child sex (*p* = 0.80), or WeeFIM scores (*p* = 0.44), but incomplete cases showed somewhat higher maternal education (*p* = 0.008) and marginally lower CBCL Total Scores (*p* = 0.053). Because missingness was associated with observed characteristics rather than occurring completely at random, the path model was estimated using full-information maximum likelihood (*n* = 79) and confirmed with multiple imputation (m = 20, Rubin’s rules); both approaches are valid under a missing-at-random assumption and use all available cases, in contrast to complete-case analysis (*n* = 45), which assumes data are missing completely at random.

In addition to the a priori power analysis described in Section 2.3, sensitivity power analyses were conducted to determine minimum detectable effect sizes for each analytical approach. These were performed in G*Power 3.1.9.7 [25]; post hoc (“observed”) power was not computed, as it is a deterministic function of the *p*-value and is uninformative [26]. All analyses assumed α = 0.05 (two-tailed) and a target power of 0.80. For the between-group comparisons (independent-samples *t*-test), with n = 79 (CP) and n = 62 (controls), the minimum detectable effect size was Cohen’s d = 0.48 (rank-biserial correlation ≈ 0.27; d = 0.55 at 0.90 power). For the within-CP associations (correlation, bivariate normal model), N = 79 yielded a minimum detectable correlation of |ρ| = 0.31 (|ρ| = 0.36 at 0.90 power), increasing to |ρ| ≈ 0.35 in the reduced subsets (n ≈ 60) affected by missing self-report data. For the covariate-adjusted models (linear multiple regression, single coefficient; four predictors, N = 141), the minimum detectable effect was f^2^ = 0.06 (standardized coefficient ≈ 0.23). The study was therefore adequately powered for medium-sized effects but underpowered for small ones, so non-significant comparisons were interpreted as inconclusive rather than as evidence for the absence of an effect. Because the parameter-to-sample-size ratio is an inadequate basis for evaluating statistical power in mediation models with resampled indirect effects, power for the indirect effects was estimated separately by Monte Carlo simulation [27] using 1000 replications and 20,000 Monte Carlo draws per replication (95% confidence intervals). For standardized paths of a = 0.41 (predictor→mediator) and b = 0.20 (mediator→outcome), power to detect the indirect effect at N = 79 was approximately 0.35, and roughly 225 dyads would be required to reach 0.80 power. The mediation analyses are therefore regarded as exploratory and hypothesis-generating.

Statistical analyses were performed using jamovi version 2.6.44 (The jamovi project, Sydney, Australia; https://www.jamovi.org, accessed on 17 June 2026) and JASP version 0.19.3 (JASP Team, Amsterdam, The Netherlands; https://jasp-stats.org, accessed on 17 June 2026). Statistical significance was set at *p* ≤ 0.05 (two-tailed) for all tests.

### 2.7. Reporting Guideline

This study was reported in accordance with the Strengthening the Reporting of Observational Studies in Epidemiology (STROBE) checklist for cross-sectional studies. The completed STROBE checklist has been provided in the Supplementary Materials.

## 3. Results

A total of 141 child–mother dyads were enrolled, comprising 79 in the CP group and 62 in the control group. The median age of children was 12.00 years [6.00–18.00] in the CP group and 9.50 years [6.00–18.00] in the control group, with a significant between-group difference (*p* = 0.027, r = −0.21). Sex distribution did not differ between groups (*p* = 0.152). Maternal age was 39.9 ± 7.41 years in the CP group and 42.3 ± 5.16 years in the control group (*p* = 0.028, d = −0.36). Large and statistically significant differences were observed for maternal education level (V = 0.75), maternal employment status (V = 0.62), paternal education level (V = 0.59), family structure (V = 0.34), and number of siblings (r = −0.63; all *p* < 0.001). The groups were comparable with respect to marital status, child educational level, and presence of concurrent illness in either parent (Table 1).

Regarding the clinical profile of the CP group, the most common subtypes were spastic (39.2%) and diplegic (27.8%). Prematurity was present in 39.2% of participants, incubator care in 58.2%, and a history of seizures in 32.9%. With respect to comprehension level, 26.6% of patients were classified as low and 40.5% as moderate. The mean WeeFIM Total score was 71.20 ± 30.5; given the possible score range of 18–126, the sample exhibited a wide functional distribution. Motor Total and Cognitive Total scores were 50.30 ± 23.90 and 20.90 ± 10.80, respectively. The IFS Total Impact score was 53.10 ± 12.90 (Table 2).

In group comparisons of scale scores, the CP group scored significantly higher than the controls on all eight CBCL syndrome subscales and both broadband scales (Internalizing and Externalizing), with the most pronounced difference observed in Social Problems. Regarding parenting variables, the PBI Care score and both PCRS subscales did not show significant between-group differences, whereas maternal Overprotection was significantly higher in the CP group. On the CERQ, mothers in the CP group scored significantly higher on Self-Blame, Acceptance, Rumination, Positive Reappraisal, and Putting into Perspective, as well as on both the adaptive and maladaptive composite scores. The remaining CERQ subscales showed no significant variations between cohorts (Table 3). In a sensitivity analysis adjusting for child age, sex, and maternal education, the CP group remained significantly higher than the controls on all CBCL outcomes (Table 4a). However, when the same covariate-adjusted model was applied to the parenting and maternal emotion-regulation outcomes, several between-group differences were not robust to demographic control. Specifically, the elevated scores in the CP group for PBI Overprotection, CERQ Self-Blame, CERQ Putting into Perspective, and the maladaptive composite attenuated to non-significance. Conversely, the differences in CERQ Acceptance, Rumination, Positive Reappraisal, and the adaptive composite remained statistically significant after adjusting for demographic factors (Table 4b).

Spearman correlations among the key variables in the CP group are presented in Table 5, and partial correlations adjusted for child age, sex, and maternal education are presented in Table 6 and Table 7. Child functional independence showed a domain-specific pattern: WeeFIM Cognitive was negatively associated with CBCL Total Problems (ρ = −0.36, *p* < 0.001), whereas WeeFIM Motor was not (ρ = −0.09, ns). WeeFIM Cognitive was also positively associated with PBI Care (ρ = 0.40, *p* < 0.01) and negatively with PBI Overprotection (ρ = −0.35, *p* < 0.01). The two parental bonding subscales related to child behavior in opposite directions: PBI Overprotection showed a moderate positive association with CBCL Total Problems (ρ = 0.53, *p* < 0.001), whereas PBI Care showed a weak negative association (ρ = −0.28, *p* < 0.05). Greater family impact was positively associated with child behavioral problems, with IFS Total Impact correlating positively with CBCL Total Problems (ρ = 0.32, *p* < 0.05). CERQ Self-Blame was positively associated with IFS Total Impact (ρ = 0.42, *p* < 0.01) but not with the PBI subscales. The adaptive and maladaptive emotion-regulation composites were strongly intercorrelated (ρ = 0.70, *p* < 0.001); the maladaptive composite was weakly positively associated with CBCL Total Problems (ρ = 0.37, *p* < 0.01), whereas the adaptive composite was not (ρ = 0.21, ns). These associations remained essentially unchanged after adjustment for child age, sex, and maternal education (Table 6 and Table 7), with the exception of the maladaptive composite–CBCL association, which attenuated to non-significance (partial ρ = 0.25, ns).

**Figure 1:**
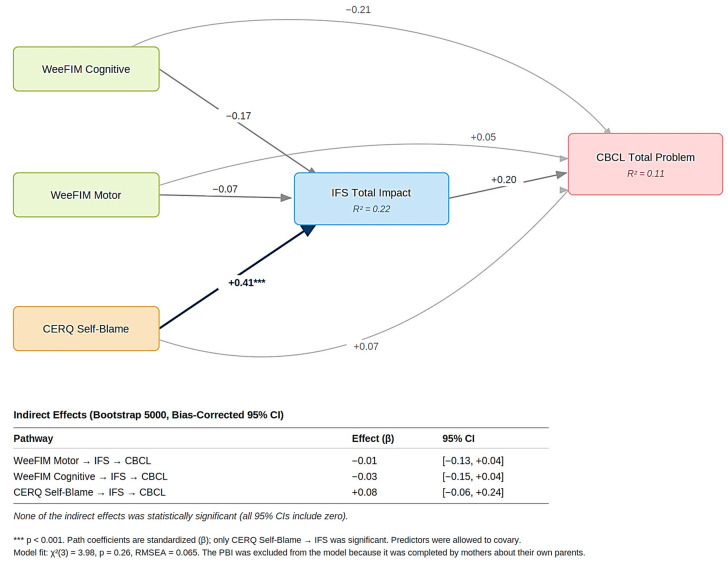
Dual pathway from functional limitations to behavioral problems in children with cerebral palsy.

In the path model (CP group, N = 79, FIML), maternal Self-Blame was the only exogenous variable significantly associated with family impact (β = 0.41, *p* < 0.001); neither WeeFIM Motor (β = −0.07, *p* = 0.55) nor WeeFIM Cognitive (β = −0.17, *p* = 0.13) was significantly associated with family impact. For child behavioral problems, family impact (β = 0.20, *p* = 0.10) and WeeFIM Cognitive (β = −0.21, *p* = 0.09) were associated in the expected directions but did not reach statistical significance, and neither WeeFIM Motor (β = 0.05, *p* = 0.68) nor Self-Blame (β = 0.07, *p* = 0.54) was significantly associated with behavioral problems. The model showed acceptable fit, χ^2^(3) = 3.98, *p* = 0.26, RMSEA = 0.065, and explained 22% of the variance in family impact and 11% in CBCL Total Problems (Figure 1, Table 8).

None of the indirect effects through family impact reached significance: WeeFIM Motor → IFS → CBCL (β = −0.01, 95% CI [−0.13, 0.04]), WeeFIM Cognitive → IFS → CBCL (β = −0.03, 95% CI [−0.15, 0.04]), and CERQ Self-Blame → IFS → CBCL (β = 0.08, 95% CI [−0.06, 0.24]). All bootstrap confidence intervals included zero, and these results were confirmed by multiple imputation (m = 20, Rubin’s rules). Accordingly, the cross-sectional data did not provide statistical support for family impact as a candidate mediator within these specific pathways. The detailed distribution of missing values across the measurement instruments and the comparison of clinical parameters between complete and incomplete cases are summarized in Table 9.

## 4. Discussion

This study evaluated the relationships linking child motor and cognitive functional independence, alongside maternal cognitive emotion regulation, to child behavioral problems in cerebral palsy, with family impact examined as a candidate mediator. In contrast to standard mediation frameworks, the statistical path analysis did not demonstrate significant indirect associations operating through family impact. Instead, the results show a direct link where child behavioral problems are associated with child cognitive functional parameters, while maternal self-blame operates as an independent factor associated with perceived family impact. These cross-sectional findings suggest that child behavioral difficulties and family-level adaptation are organized through distinct parallel systems rather than a single interconnected pathway.

Regarding the child’s functional parameters, the correlation analysis points to an association between lower child cognitive independence and higher behavioral problem scores, a trend that remained consistent within the structural path model. These cross-sectional relationships are consistent with the clinical reality that reduced communication skills and limited reciprocal interaction opportunities in children with lower cognitive functional independence are closely linked with elevated behavioral difficulties. While this structure conceptually complements the work of Leader et al. [28], who observed high rates of behavioral problems in children with cerebral palsy and identified intellectual disability as a relevant clinical factor, it is important to emphasize that the WeeFIM metrics capture everyday functional communication and social cognition rather than innate intellectual capacity or IQ profiles. Furthermore, this pattern is consistent with clinical observations suggesting that when a child faces severe cognitive and communication limitations, the daily mother–child dynamic frequently moves toward a unilateral, caregiving-dominated structure, which may narrow the space for adaptive emotional regulation.

The robust association between maternal Self-Blame and perceived family impact in our path model suggests that maternal cognitive emotion regulation capacity is linked with family-level adaptation independently of the child’s objective functional status. The near-zero correlation observed between the WeeFIM parameters and maternal CERQ scores raises the possibility that maternal self-blame may reflect a relatively stable cognitive-emotional tendency rather than a direct response to the child’s current functional status. However, as a cross-sectional design cannot establish temporal precedence, this interpretation remains hypothesis-generating; self-blame could equally represent a reaction to earlier events (e.g., diagnosis, perceived stigma, or unmeasured aspects of caregiving burden) that are not captured by current functional indices. Longitudinal designs tracking maternal cognitive emotion regulation from diagnosis onward are needed to determine whether self-blame precedes, co-occurs with, or develops in response to the child’s evolving functional and behavioral profile. This framework is consistent with the model of Dix [29], who characterized parenting as an emotionally organized process, and with Morris et al. [6], who suggested that parental emotion regulation capacity is closely connected with the emotional climate of the family. This view also aligns with Hajal and Paley [7], who integrated parenting behaviors with the parent’s own internal emotional processes. Within this context, maternal self-blame is associated with amplified feelings of inadequacy and fear of making mistakes, which may partition family adaptation along more stressful lines. Elevated self-criticism in mothers of children with CP is consistent with more pronounced unresolved emotional processes [9], and empirical evidence suggests that guilt in mothers of children with developmental disabilities is associated with broader psychosocial outcomes [30]. Furthermore, the link showing that maternal self-blame and catastrophizing are associated with adverse outcomes in adolescents through the adolescent’s own maladaptive emotion regulation strategies [31] suggests that this mechanism is a general systemic factor rather than a feature specific to cerebral palsy.

Regarding the physical dimensions of the model, the path analysis showed that child motor independence demonstrated a weak negative association with family impact parameters, a pattern that did not reach statistical significance. Consequently, our cross-sectional data did not provide statistical support for family impact as a significant intervening mechanism between child motor capacity and child behavioral difficulties. Systematic review evidence identifies the severity of physical disability as an important factor associated with caregiving burden [6], and empirical data suggest that the links involving caregiving burden and broader family outcomes are characterized by complex mechanisms [32]. The absence of a strong statistical association between child motor scores and family impact in our sample suggests that caregiving demands in cerebral palsy may present different qualitative challenges across varying levels of physical disability. While severe motor impairment requires physical organization around basic daily care, families of children with higher motor functioning may navigate different adaptation challenges related to broader social integration and expectation management. These cross-sectional patterns suggest that child motor trajectories and family-level adjustments are complex systems where functional gains do not automatically translate into a linear reduction in perceived family impact.

The prominent positive association between maternal Self-Blame and perceived family impact in the path model requires careful clinical interpretation. One might expect that a maladaptive strategy such as self-blame exacerbates the psychological toll on the caregiver; our cross-sectional findings support this perspective by indicating that mothers who employ higher levels of self-blame also report a significantly greater impact on the family system. This pattern suggests that when a mother processes caregiving difficulties within a framework of personal shortcomings and internalized responsibility, this cognitive stance is closely linked with an amplified perception of global family burden. Rather than sequestering the stress within her internal evaluative domain, maternal self-blame appears to be a systemic factor that covaries with broader difficulties in family adaptation. This interconnectedness points to the clinical relevance of identifying and screening maternal cognitive distortions during child rehabilitation, as these individual cognitive appraisals are tightly woven into the perceived welfare of the entire family structure.

Regarding the final parameters of the model, the correlation analysis points to a positive association between higher perceived family impact and elevated child behavioral problems, a trend that remained consistent within the path analysis framework. This positive direction aligns with general clinical expectations, suggesting that greater psychosocial and financial strain within the family environment covaries with more pronounced behavioral and emotional difficulties in the child. The shared variance between these family-level adaptation challenges and child psychopathology underscores the systemic nature of chronic caregiving conditions in cerebral palsy, where family burden and child behavioral outcomes show closely interconnected trajectories. These cross-sectional patterns suggest that family-focused support strategies may be relevant components when managing child behavior problems, as the difficulties within the family ecology are tightly associated with the child’s emotional and behavioral presentation.

Group comparisons provide clinical context for the structural path model findings. Higher CBCL scores across multiple domains in children with CP relative to controls are consistent with both foundational and recent evidence indicating elevated emotional, behavioral, attentional, and social difficulties in this population [2,3,33]. The persistence of attention and social interaction difficulties across development is consistent with the view that these challenges may follow a chronic trajectory into adulthood [34,35]. Regarding parenting attitudes, the unadjusted analysis indicated higher maternal overprotection in the CP group; however, our sensitivity analysis showed that this difference was not robust against demographic imbalances, suggesting that parenting variations may be closely intertwined with socioeconomic and maternal educational characteristics rather than the child’s clinical status alone [36,37]. The simultaneous elevation of adaptive and maladaptive coping strategies among mothers of children with CP suggests that strain and adaptation processes coexist under chronic caregiving conditions, as observed in the recent literature on caregiver burden and psychosocial adjustment in CP [38,39]. Given the robust association between maladaptive emotion regulation strategies and psychopathology reported in the meta-analytic literature [10,40], this pattern warrants clinical attention. Demographic imbalances between the study groups require a cautious interpretation of the univariate findings; however, because the structural path analysis was conducted exclusively within the CP group, these differences do not directly partition the internal relations tested in the path model.

That maternal self-blame is associated with family dynamics independently of the child’s functional level suggests that further exploring these cognitive emotion regulation patterns as prospective targets for future screening and longitudinal research within CP rehabilitation may be valuable. Rather than providing definitive evidence for immediate therapeutic integration, these cross-sectional findings serve a hypothesis-generating role, pointing to maternal self-blame as a potential target for future intervention trials to evaluate its causal impact on family adaptation and child behavior. Mothers of children with lower cognitive functional independence may constitute a particularly vulnerable subgroup with respect to adverse child behavioral outcomes and subsequent family adaptation challenges. A recent study conducted within the same clinical and research context utilizing an independent cohort indicated that maternal cognitive-emotional factors, such as repressed anger, are closely linked with behavioral problems in children with CP through caregiver distress frameworks [41], providing further support for this family-centered approach. Beyond maternal-focused interventions, family-centered rehabilitation programs may also benefit from actively encouraging paternal involvement in daily caregiving and therapeutic routines. A more equitable distribution of caregiving responsibilities between parents could reduce maternal burden and potentially attenuate the self-blame processes identified in this study; future research should examine whether paternal engagement moderates the relationship between maternal cognitive emotion regulation and family adaptation outcomes in CP.

### Strengths, Limitations, and Future Directions

Among the strengths of this study are the evaluation of an integrated model testing the cognitive, physical, and maternal cognitive-emotional factors simultaneously within the CP field, the data-driven model evaluation with bootstrap-based indirect association testing, and the combined use of clinician-rated WeeFIM and self-report measures, which partially reduces informant bias.

Several limitations should be acknowledged. The cross-sectional design precludes a direct evaluation of temporal precedence among variables; the model does not provide evidence of causation but rather suggests the fit of a theoretically grounded relational structure with the data. Our sample size (*n* = 79 in the CP group) restricted the complexity of the path analysis. A Monte Carlo power analysis further indicated low statistical power (approximately 0.35) to detect indirect (mediated) effects of the magnitude observed here, with roughly 225 dyads required to reach 0.80 power; the non-significant and borderline indirect effects should therefore be regarded as inconclusive (possible Type II error) rather than as evidence against mediation, and require confirmation in larger, prospective samples with a more homogeneous age distribution. Although we adjusted for key demographic confounders (child age, sex, and maternal education) in our preliminary sensitivity analyses and partial correlations, the limited sample size precluded the direct inclusion of additional clinical and sociodemographic covariates (e.g., CP subtype, epilepsy, intellectual disability, maternal employment, and socioeconomic status) into the final path model to prevent overfitting and the loss of statistical power. Future large-cohort studies are needed to evaluate the independent relational structures of these specific clinical parameters within the proposed framework. Furthermore, the single-center setting and the reliance on data from a single cultural context limit the generalizability, and replication across diverse clinical contexts is needed. Additionally, the lack of documentation regarding whether the participating mothers received professional psychological support, as well as the specific type of full-time school enrollment for the children, introduces a risk of unmeasured confounding in our findings and represents a constraint that should be addressed in future trials. The reliance of most instruments on maternal self-report carries common method variance risk, although the clinician-rated WeeFIM partially mitigates this concern. To provide a more comprehensive validation, future studies should consider multi-informant designs incorporating teacher reports, direct clinical observations, or child self-reports where feasible, thereby minimizing individual response biases and capturing behavioral variations across different environmental contexts. The lack of WeeFIM and IFS administration to the control group necessitated testing the structural model within the CP group only. Finally, another limitation is the absence of systematic standardized classifications for CP severity, such as GMFCS, MACS, or CFCS, which could not be retrospectively reconstructed from clinical records and should be included in future studies to better stratify functional and behavioral outcomes. Although formal severity classification was unavailable, several severity-related clinical indicators were systematically recorded and are reported in Table 2, including seizure history (32.9%), visual and hearing impairments (21.5% and 6.3%, respectively), comprehension and swallowing levels, and the extent of rehabilitation and special education support (91.1% and 77.2%, respectively). Together, these parameters demonstrate a clinically heterogeneous sample with a substantial proportion of moderate-to-severe involvement, providing indirect contextual information for interpreting the functional and behavioral findings, even though sample size constraints precluded their direct inclusion as covariates in the path model.

Future studies should evaluate the temporal characteristics of these associations through longitudinal designs, conduct subgroup analyses using multicenter cohorts with systematic GMFCS documentation, and examine the potential of cognitive intervention programs targeting maternal self-blame to improve family adaptation and child behavioral outcomes through randomized controlled trials. In conclusion, this study contributes to the literature by providing an integrated evaluation of the interconnected roles of family ecology and the independent relevance of maternal cognitive emotion regulation within the framework of child functional parameters and behavioral difficulties in cerebral palsy. These cross-sectional findings suggest that comprehensive cerebral palsy rehabilitation paradigms may benefit from encompassing the evaluation of maternal cognitive emotion regulation profiles alongside child functional capacity.

## 5. Conclusions

In conclusion, this study suggests that behavioral problems in children with cerebral palsy and family-level adaptation challenges are organized through distinct, parallel mechanisms rather than a single interconnected causal pathway. Child behavioral outcomes show a direct association with child cognitive functional parameters, whereas maternal self-blame operates as a robust, independent factor tightly linked with perceived family impact, regardless of the child’s objective motor or cognitive functional independence. These cross-sectional findings identify maternal cognitive emotion regulation, particularly maternal self-blame, as a potentially relevant psychosocial construct that warrants further investigation in future longitudinal and intervention studies rather than as an established therapeutic target. Future longitudinal and randomized intervention studies are needed to determine whether screening for maternal self-blame or interventions targeting maternal cognitive emotion regulation improve family adaptation or child behavioral outcomes.

## Figures and Tables

**Table 1:** Demographic characteristics of the study groups.

Variables	CP Group (*n* = 79)	Control Group (*n* = 62)	*p*	Effect Size
**Child’s age (years) †**	12.00 [6.00–18.00]	9.50 [6.00–18.00]	**0.027**	r = −0.21
**Maternal age (years) §**	39.9 ± 7.41	42.3 ± 5.16	**0.028**	d = −0.36
**Sex**			0.152	V = 0.12
Female	44 (55.7)	27 (43.5)		
Male	35 (44.3)	35 (56.5)		
**Marital status ‡**			0.242	V = 0.17
Married	74 (93.7)	52 (83.9)		
Single	0 (0.0)	1 (1.6)		
Divorced	4 (5.1)	8 (12.9)		
Widowed	1 (1.3)	1 (1.6)		
**Maternal employment**, employed	12 (15.2)	48 (77.4)	**<0.001**	V = 0.62
**Maternal education**			**<0.001**	V = 0.75
Primary school	15 (19.0)	1 (1.6)		
Secondary school	16 (20.3)	0 (0.0)		
High school	31 (39.2)	3 (4.8)		
University	16 (20.3)	30 (48.4)		
Master’s degree	1 (1.3)	17 (27.4)		
Doctoral degree	0 (0.0)	11 (17.7)		
**Paternal education ‡**			**<0.001**	V = 0.59
Primary school	11 (13.9)	0 (0.0)		
Secondary school	8 (10.1)	2 (3.2)		
High school	39 (49.4)	7 (11.3)		
University	17 (21.5)	37 (59.7)		
Master’s degree	4 (5.1)	13 (21.0)		
Doctoral degree	0 (0.0)	3 (4.8)		
**Number of siblings †**	2.00 [0.00–8.00]	1.00 [0.00–2.00]	**<0.001**	r = −0.63
**Family structure**			**<0.001**	V = 0.34
Nuclear family	61 (77.2)	52 (83.9)		
Extended family	16 (20.3)	0 (0.0)		
Single-parent family	2 (2.5)	10 (16.1)		
**Delivery mode**			**0.027**	V = 0.18
Vaginal delivery	36 (45.6)	17 (27.4)		
Cesarean section	43 (54.4)	45 (72.6)		
**Child’s education level ‡**			0.414	V = 0.11
Primary school	46 (58.2)	33 (53.2)		
Secondary school	23 (29.1)	16 (25.8)		
High school	10 (12.7)	13 (21.0)		
**Maternal comorbidity**, present	11 (13.9)	8 (8.9)	0.718	V = 0.03
**Paternal comorbidity**, present	7 (9.5)	4 (6.5)	0.522	V = 0.05

Continuous variables are presented as mean ± SD or median [minimum–maximum] according to normality (Shapiro–Wilk test). Categorical variables are presented as n (%). Effect sizes: Cohen’s d [95% CI] for parametric comparisons, rank-biserial correlation for nonparametric comparisons, Cramér’s V for categorical comparisons. † Mann–Whitney U test; ‡ Fisher–Freeman–Halton exact test; § Welch *t*-test. Unmarked continuous variables were compared using Student’s *t*-test; unmarked categorical variables were compared using the Pearson chi-square test. Bold *p*-values indicate statistical significance (*p* ≤ 0.05).

**Table 2:** Clinical characteristics, WeeFIM scores, and IFS scores of the CP group.

Variables	n (%) or Mean ± SD
**CP type**	
Spastic	31 (39.2)
Diplegic	22 (27.8)
Hemiplegic	9 (11.4)
Ataxic	7 (8.9)
Tetraplegic	4 (5.1)
Mixed	4 (5.1)
Athetoid	2 (2.5)
**Prematurity**, present	31 (39.2)
**Incubator care**, present	46 (58.2)
**Neonatal jaundice**, present	31 (39.2)
**Seizure history**, present	26 (32.9)
**Comprehension level**	
High	26 (32.9)
Moderate	32 (40.5)
Low	21 (26.6)
**Speech level**	
High	26 (32.9)
Moderate	35 (44.3)
Low	18 (22.8)
**Swallowing level**	
High	50 (63.3)
Moderate	24 (30.4)
Low	5 (6.3)
**Visual impairment**, present	17 (21.5)
**Hearing impairment**, present	5 (6.3)
**Incontinence**, present	11 (13.9)
**Special education**, receiving	61 (77.2)
**Rehabilitation support**, receiving	72 (91.1)
**Home care support**, present	18 (22.8)
**Psychological support**, receiving	8 (10.1)
**Botulinum toxin/orthopedic surgery**, applied	34 (43.0)
**Age at CP diagnosis** (years)	1.00 [0.00–9.00]
**WeeFIM subscales**	
Self-care (6–42)	22.40 ± 12.30
Sphincter control (2–14)	8.61 ± 4.74
Transfer (3–21)	11.20 ± 6.10
Locomotion (2–14)	9.19 ± 4.58
Communication (2–14)	9.08 ± 4.39
Social cognition (3–21)	12.00 ± 6.74
Motor total (13–91)	50.30 ± 23.90
Cognitive total (5–35)	20.90 ± 10.80
Total score (18–126)	71.20 ± 30.50
**IFS subscales**	
Financial burden (4–16)	8.66 ± 2.50
Familial/social impact (9–36)	24.40 ± 6.36
Personal strain (6–24)	20.04 ± 6.20
Mastery (5–20)	11.9 ± 2.79
Total impact (19–76)	53.10 ± 12.90

Continuous variables are presented as mean ± SD or median [minimum–maximum]. Categorical variables are presented as n (%). WeeFIM: Functional Independence Measure for Children; IFS: Impact on Family Scale.

**Table 3:** Comparison of scale scores between study groups.

Variables	CP Group (*n* = 79)	Control Group (*n* = 62)	*p*	Effect Size [95% CI]
**CBCL Syndrome Scales**				
Anxious/Depressed †	6.0 [0–22]	3.0 [0–14]	<0.001	r = 0.40 [0.22, 0.56]
Withdrawn/Depressed †	3.0 [0–11]	0.0 [0–9]	<0.001	r = 0.54 [0.38, 0.69]
Somatic Complaints †	3.0 [0–19]	1.0 [0–12]	<0.001	r = 0.45 [0.27, 0.61]
Social Problems †	7.0 [0–17]	1.0 [0–12]	<0.001	r = 0.64 [0.48, 0.78]
Thought Problems †	5.0 [0–18]	1.0 [0–13]	<0.001	r = 0.56 [0.40, 0.70]
Attention Problems †	6.0 [0–15]	2.0 [0–12]	<0.001	r = 0.60 [0.44, 0.74]
Rule-Breaking Behavior †	4.0 [0–23]	2.0 [0–6]	<0.001	r = 0.53 [0.37, 0.68]
Aggressive Behavior †	8.0 [0–24]	2.5 [0–15]	<0.001	r = 0.54 [0.38, 0.68]
**CBCL Broadband Scales**				
Internalizing †	15.0 [0–47]	5.0 [0–35]	<0.001	r = 0.51 [0.36, 0.67]
Externalizing †	12.0 [0–47]	4.0 [0–19]	<0.001	r = 0.57 [0.41, 0.71]
**PBI**				
Care †	24.0 [4–35]	24.0 [0–36]	0.969	r = 0.00 [−0.21, 0.19]
Overprotection	16.41 ± 6.62	12.71 ± 6.56	0.002	d = 0.56 [0.20, 0.92]
**PCRS**				
Positive Subscale †	41.0 [22–50]	41.0 [34–50]	0.855	r = −0.02 [−0.23, 0.19]
Negative Subscale †	15.0 [4–25]	15.0 [7–20]	0.880	r = 0.02 [−0.20, 0.22]
**CERQ Subscales**				
Self-Blame	11.69 ± 3.71	10.44 ± 2.62	0.032	d = 0.39 [0.03, 0.75]
Acceptance †	13.0 [3–20]	11.0 [6–16]	0.011	r = 0.26 [0.06, 0.47]
Rumination †	13.0 [4–20]	12.0 [7–19]	0.030	r = 0.23 [0.02, 0.42]
Positive Refocusing †	13.0 [2–20]	12.0 [6–18]	0.115	r = 0.16 [−0.04, 0.36]
Refocus on Planning	13.87 ± 3.74	13.53 ± 3.30	0.597	d = 0.10 [−0.26, 0.45]
Positive Reappraisal	13.20 ± 3.79	11.32 ± 2.92	0.003	d = 0.55 [0.19, 0.92]
Putting into Perspective	9.49 ± 4.03	7.29 ± 2.33	<0.001	d = 0.67 [0.30, 1.04]
Catastrophizing †	9.0 [1–18]	8.0 [4–14]	0.100	r = 0.17 [−0.04, 0.38]
Other-Blame †	9.0 [3–18]	8.0 [4–14]	0.483	r = 0.07 [−0.15, 0.29]
**CERQ Composite Scores**				
Adaptive Total †	63.0 [12–83]	55.5 [37–82]	0.001	r = 0.34 [0.15, 0.53]
Maladaptive Total †	42.0 [22–73]	39.0 [25–56]	0.038	r = 0.22 [0.01, 0.42]

Note. Continuous variables are presented as mean ± SD (normal distribution) or median [minimum–maximum] (non-normal distribution). † Mann–Whitney U test. Unmarked variables were compared using Student’s *t*-test. Effect sizes: Cohen’s d [95% CI] for parametric comparisons; rank-biserial correlation [95% CI] for nonparametric comparisons. CBCL: Child Behavior Checklist; PBI: Parental Bonding Instrument; PCRS: Parent–Child Relationship Scale; CERQ: Cognitive Emotion Regulation Questionnaire.

**Table 4:** (**a**) Sensitivity analysis: between-group differences in CBCL outcomes adjusted for child age, sex, and maternal education. (**b**) Sensitivity analysis: between-group differences in parenting (PBI, PCRS) and maternal cognitive emotion-regulation (CERQ) outcomes adjusted for child age, sex, and maternal education.

(**a**)			
**CBCL Outcome**	**Unadjusted SMD**	**Adjusted β [95% CI]**	***p***
CBCL Total Problems	+1.01	**+0.83 [+0.44, +1.22]**	<0.001
Internalizing	+0.85	**+0.70 [+0.26, +1.13]**	0.002
Externalizing	+0.93	**+0.77 [+0.37, +1.17]**	<0.001
Anxious/Depressed	+0.67	**+0.62 [+0.16, +1.09]**	0.008
Withdrawn/Depressed	+0.91	**+0.73 [+0.32, +1.14]**	<0.001
Somatic Complaints	+0.75	**+0.61 [+0.19, +1.03]**	0.004
Social Problems	+1.09	**+0.90 [+0.48, +1.32]**	<0.001
Thought Problems	+0.89	**+0.78 [+0.37, +1.18]**	<0.001
Attention Problems	+1.06	**+0.82 [+0.46, +1.19]**	<0.001
Rule-Breaking	+0.85	**+0.73 [+0.33, +1.12]**	<0.001
Aggressive	+0.91	**+0.74 [+0.33, +1.15]**	<0.001
(**b**)			
**Outcome**	**Unadjusted SMD**	**Adjusted β [95% CI]**	***p***
**PBI**			
Care	+0.05	+0.18 [−0.32, +0.68]	0.485
Overprotection	+0.54	+0.27 [−0.23, +0.77]	0.293
**PCRS**			
Positive Subscale	−0.04	−0.37 [−0.94, +0.20]	0.206
Negative Subscale	−0.06	−0.03 [−0.56, +0.50]	0.917
**CERQ Subscales**			
Self-Blame	+0.38	+0.32 [−0.18, +0.82]	0.214
Acceptance	+0.34	**+0.93 [+0.47, +1.40]**	<0.001
Rumination	+0.36	**+0.60 [+0.07, +1.14]**	0.027
Positive Refocusing	+0.28	+0.35 [−0.18, +0.88]	0.197
Refocus on Planning	+0.10	+0.18 [−0.42, +0.78]	0.553
Positive Reappraisal	+0.54	**+0.71 [+0.20, +1.22]**	0.006
Putting into Perspective	+0.64	+0.43 [−0.06, +0.93]	0.087
Catastrophizing	+0.32	+0.09 [−0.45, +0.64]	0.736
Other-Blame	+0.18	−0.03 [−0.62, +0.56]	0.925
**CERQ Composite Scores**			
Adaptive Total	+0.52	**+0.70 [+0.22, +1.18]**	0.005
Maladaptive Total	+0.47	+0.29 [−0.20, +0.77]	0.246

Note. Each CBCL outcome was standardized and regressed on group (CP vs. control), child age, child sex, and maternal education using ordinary least squares with heteroscedasticity-consistent (HC3) standard errors. Unadjusted SMD = standardized mean difference (CP − control); Adjusted β = standardized group difference after covariate adjustment. Positive values indicate higher scores in the CP group. CBCL: Child Behavior Checklist. Bold adjusted coefficients are significant at *p* < 0.05. PBI: Parental Bonding Instrument; PCRS: Parent–Child Relationship Scale; CERQ: Cognitive Emotion Regulation Questionnaire.

**Table 5:** Zero-order Spearman correlations among study variables (CP group, *n* = 79).

Variable	1	2	3	4	5	6	7	8	9	10	11
1. WeeFIM Motor	—										
2. WeeFIM Cognitive	0.47 ***	—									
3. PBI Care	0.15	0.40 **	—								
4. PBI Overprotection	0.02	−0.35 **	−0.58 ***	—							
5. IFS Total Impact	−0.18	−0.25 *	−0.36 **	0.33 *	—						
6. CERQ Self-Blame	−0.06	−0.03	−0.18	0.10	0.42 **	—					
7. CERQ Adaptive	−0.13	−0.08	−0.15	−0.07	0.38 **	0.71 ***	—				
8. CERQ Maladaptive	−0.02	−0.06	−0.18	0.25	0.39 **	0.81 ***	0.70 ***	—			
9. CBCL Internalizing	−0.07	−0.26 *	−0.24	0.44 ***	0.25	0.30 *	0.35 **	0.40 **	—		
10. CBCL Externalizing	−0.09	−0.34 **	−0.27 *	0.47 ***	0.22	0.10	0.16	0.27 *	0.78 ***	—	
11. CBCL Total Problems	−0.09	−0.36 ***	−0.28 *	0.53 ***	0.32 *	0.21	0.21	0.37 **	0.92 ***	0.93 ***	—

Note. Spearman ρ. * *p* < 0.05, ** *p* < 0.01, *** *p* < 0.001. Significant coefficients are shown in bold. Pairwise n varies (60–79) due to missing data. Effect sizes are associations, not causal effects.

**Table 6:** Partial Spearman correlations adjusted for child age, sex, and maternal education (CP group).

Variable	1	2	3	4	5	6	7	8	9	10	11
1. WeeFIM Motor	—										
2. WeeFIM Cognitive	0.47 ***	—									
3. PBI Care	0.12	0.36 **	—								
4. PBI Overprotection	0.01	−0.36 **	−0.58 ***	—							
5. IFS Total Impact	−0.15	−0.25	−0.36 *	0.29 *	—						
6. CERQ Self-Blame	−0.02	0.09	−0.10	0.10	0.37 *	—					
7. CERQ Adaptive	−0.10	−0.02	−0.08	−0.09	0.30	0.66 ***	—				
8. CERQ Maladaptive	0.07	0.04	−0.12	0.24	0.29	0.79 ***	0.66 ***	—			
9. CBCL Internalizing	−0.05	−0.23	−0.23	0.42 **	0.20	0.19	0.25	0.29 *	—		
10. CBCL Externalizing	−0.08	−0.31 **	−0.25	0.45 ***	0.17	−0.01	0.06	0.16	0.77 ***	—	
11. CBCL Total Problems	−0.07	−0.33 **	−0.25 *	0.52 ***	0.26 *	0.08	0.08	0.25	0.91 ***	0.93 ***	—

Note. Partial Spearman ρ controlling for child age, child sex, and maternal education level. * *p* < 0.05, ** *p* < 0.01, *** *p* < 0.001. Significant coefficients in bold.

**Table 7:** Key path-model associations: zero-order vs. adjusted partial Spearman correlations with 95% CI (CP group).

Association	Zero-Order ρ [95% CI]	Adjusted ρ [95% CI]
WeeFIM Motor—IFS Total Impact	−0.18 [−0.41, 0.07]	−0.15 [−0.39, 0.11]
WeeFIM Cognitive—PBI Care	0.40 [0.17, 0.58] **	0.36 [0.12, 0.56] **
WeeFIM Cognitive—PBI Overprotection	−0.35 [−0.55, −0.11] **	−0.36 [−0.56, −0.11] **
IFS Total Impact—CBCL Total	0.32 [0.07, 0.53] *	0.26 [0.01, 0.49] *
PBI Care—CBCL Total	−0.28 [−0.49, −0.04] *	−0.25 [−0.48, −0.00] *
PBI Overprotection—CBCL Total	0.53 [0.33, 0.69] ***	0.52 [0.30, 0.68] ***
CERQ Self-Blame—IFS Total Impact	0.42 [0.15, 0.64] **	0.37 [0.08, 0.61] *
CERQ Self-Blame—PBI Care	−0.18 [−0.44, 0.11]	−0.10 [−0.38, 0.20]
CERQ Self-Blame—PBI Overprotection	0.10 [−0.20, 0.38]	0.10 [−0.21, 0.39]
CERQ Adaptive—CBCL Total	0.21 [−0.05, 0.44]	0.08 [−0.18, 0.33]
CERQ Maladaptive—CBCL Total	0.37 [0.12, 0.57] **	0.25 [−0.01, 0.48]
WeeFIM Cognitive—CBCL Total	−0.36 [−0.54, −0.15] ***	−0.33 [−0.52, −0.11] **
WeeFIM Motor—CBCL Total	−0.09 [−0.30, 0.14]	−0.07 [−0.29, 0.16]

Note. Adjusted = partial Spearman ρ controlling for child age, child sex, and maternal education. * *p* < 0.05, ** *p* < 0.01, *** *p* < 0.001. Associations are cross-sectional and non-causal.

**Table 8:** Path analysis: standardized direct and indirect effects on CBCL Total Problems (CP group, *n* = 79).

**Panel A. Direct Effects (Standardized)**	**β**	**SE**	***p***	**95% CI**	**R^2^**
Family impact (IFS Total Impact)					0.22
WeeFIM Motor	−0.07	0.11	0.546	[−0.29, +0.15]	
WeeFIM Cognitive	−0.17	0.11	0.132	[−0.39, +0.05]	
CERQ Self-Blame	+0.41	0.10	<0.001	[+0.21, +0.61]	
CBCL Total Problems					0.11
WeeFIM Motor	+0.05	0.12	0.681	[−0.19, +0.29]	
WeeFIM Cognitive	−0.21	0.12	0.085	[−0.45, +0.03]	
IFS Total Impact	+0.20	0.12	0.100	[−0.04, +0.43]	
CERQ Self-Blame	+0.07	0.12	0.543	[−0.16, +0.30]	
**Panel B. Indirect Effects (Standardized)**	**β**			**95% CI**	
WeeFIM Motor → IFS → CBCL	−0.01			[−0.13, +0.04]	
WeeFIM Cognitive → IFS → CBCL	−0.03			[−0.15, +0.04]	
CERQ Self-Blame → IFS → CBCL	+0.08			[−0.06, +0.24]	

Note. Table 8 exclusively reports the estimated direct and indirect effects of the structural path model. Observed-variable path analysis estimated by full-information maximum likelihood; standardized coefficients (β) reported. Panel A: direct effects with 95% confidence intervals. Panel B: indirect effects with bootstrap 95% confidence intervals; intervals containing zero are non-significant. Model fit: χ^2^(3) = 3.98, *p* = 0.26, RMSEA = 0.065. The PBI was excluded from the model because it was completed by mothers about their own parents. IFS: Impact on Family Scale; CBCL: Child Behavior Checklist; CERQ: Cognitive Emotion Regulation Questionnaire. However, because the Monte Carlo power analysis indicated that the study was underpowered to detect indirect effects of this magnitude (power ≈ 0.35; see Statistical Analysis), these mediation findings are interpreted cautiously and treated as exploratory; the non-significant indirect effects should be read as inconclusive rather than as evidence against mediation.

**Table 9:** Missing data and comparison of complete versus incomplete cases (CP group, *n* = 79).

**Panel A. Missing Data by Instrument**	**Available n**	**Missing n (%)**	
WeeFIM Motor (clinician-rated)	79	0 (0.0)	
WeeFIM Cognitive (clinician-rated)	79	0 (0.0)	
CBCL Total Problems	79	0 (0.0)	
IFS Total Impact	62	17 (21.5)	
PBI Care	64	15 (19.0)	
PBI Overprotection	63	16 (20.3)	
PCRS	62	17 (21.5)	
CERQ subscales	61	18 (22.8)	
**Panel B. Complete vs. Incomplete Cases**	**Complete** (*n* = 45)	**Incomplete** (*n* = 34)	***p***
Child age, median	11.0	12.0	0.42
Child sex	—	—	0.80
Maternal education, median	2.0	3.0	0.008
WeeFIM Total, median	73.0	70.5	0.44
CBCL Total, median	54.0	45.5	0.053

Note. Panel A: extent of missing data per instrument in the CP group; clinician-rated WeeFIM and CBCL had no missing values, whereas maternal self-report instruments had ~19–23% missing. Panel B: complete versus incomplete cases (with respect to the path-model variables) compared with the Mann–Whitney U test (continuous) or χ^2^ test (sex). Missingness was associated with maternal education but not with child age, sex, or WeeFIM scores. Accordingly, the path model used full-information maximum likelihood and multiple imputation rather than complete-case analysis.

## Data Availability

The data presented in this study are available on reasonable request from the corresponding author. The data are not publicly available due to privacy and ethical restrictions.

## Supplementary Materials

The following supporting information can be downloaded at: https://www.mdpi.com/article/10.3390/healthcare14142038/s1, Table S1: STROBE Statement—Checklist of Items for Cross-Sectional Studies.
