# Supplementary material for: Cross-Sectional Associations Between Functional Independence and Behavioral Problems in Children with Cerebral Palsy: The Relational Roles of Family Impact and Maternal Self-Blame
Source: Healthcare (Basel). 2026 Jul 8;14(14):2038. doi: 10.3390/healthcare14142038 (PMC13409949; doi:10.3390/healthcare14142038)
Supplement: Supplementary file 1 [file healthcare-14-02038-s001.zip › healthcare-4330969-supplementary.pdf]

STROBE Statement — Checklist of Items for Cross-Sectional Studies

Cross-Sectional Associations Between Functional Independence and Behavioral Problems in Children with Cerebral Palsy: The Relational Roles of Family Impact and Maternal Self-Blame

* Give information separately for cases and non-cases (exposed/unexposed) where applicable.

Item	No	Recommendation	Reported in manuscript / location
**Title and abstract**			
	1a	Indicate the study's design with a commonly used term in the title or the abstract	Title and Abstract: The terms "cross-sectional" and "observational-analytic" appear in the abstract and in Section 2.1.
	1b	Provide in the abstract an informative and balanced summary of what was done and what was found	Abstract: Background, objectives, methods, results, and conclusions are summarized. Specific statistical values removed per Reviewer 2 recommendation; narrative summary provided.
**Introduction**			
Background / rationale	2	Explain the scientific background and rationale for the investigation being reported	Introduction, paragraphs 1–5: prevalence of CP, behavioral problems, caregiving burden, maternal emotion regulation, and rationale for integrated modeling.
Objectives	3	State specific objectives, including any prespecified hypotheses	Introduction, final paragraph: primary aim (path model examining associations among WeeFIM domains, maternal self-blame, IFS, and CBCL) and secondary aim (group comparisons with sensitivity analyses) stated explicitly.
**Methods**			
Study design	4	Present key elements of study design early in the paper	Section 2.1: cross-sectional, prospective, single-center, observational-analytic design stated. Recruitment period January 2025–January 2026.
Setting	5	Describe the setting, locations, and relevant dates, including periods of recruitment, exposure, follow-up, and data collection	Section 2.1: outpatient and inpatient early intervention and rehabilitation services in Istanbul, Turkey. Recruitment period January 2025–January 2026. Data collection was cross-sectional (single time point).
Participants	6a	Give the eligibility criteria, and the sources and methods of selection of participants	Section 2.3: CP group — confirmed CP diagnosis (SCPE criteria), aged 6–18 years, maternal literacy required; comorbid psychiatric diagnoses excluded. Control group — typically developing children from well-child outpatient unit, same institution; chronic/neurological/psychiatric conditions excluded. Consecutive sampling applied to both groups.
Variables	7	Clearly define all outcomes, exposures, predictors, potential confounders, and effect modifiers. Give diagnostic criteria, if applicable	Section 2.5 (Variable Definitions and Endpoints): CBCL Total Problems = primary outcome; WeeFIM Motor and Cognitive = independent variables; IFS Total Impact = candidate mediator;

Item	No	Recommendation	Reported in manuscript / location
			CERQ Self-Blame = exogenous variable. Confounders (child age, sex, maternal education) defined and adjusted for in sensitivity analyses and partial correlations.
Data sources / measurement	8*	For each variable of interest, give sources of data and details of methods of assessment. Describe comparability of assessment methods if more than one group	Section 2.4 (Instruments): all six instruments described with psychometric references, Turkish adaptation details, item ranges, and scoring directions. WeeFIM and IFS administered only to CP group (rationale provided for each). CBCL, PBI, PCRS, and CERQ administered to both groups.
Bias	9	Describe any efforts to address potential sources of bias	Section 2.6 (Statistical Analysis): demographic imbalances addressed via HC3-robust OLS sensitivity analyses (Tables 4a/4b). FIML used to avoid complete-case bias due to ~20% missingness on self-report instruments. Clinician-rated WeeFIM partially mitigates common-method variance. Remaining bias sources discussed in Section 4.1.
Study size	10	Explain how the study size was arrived at	Section 2.3 (Participants and Sample Size Justification): a priori power analysis for group comparisons using G*Power 3.1 (Cohen's $d = 0.55$, $\alpha = 0.05$, $1-\beta = 0.80$ → minimum 53/group; +15% attrition → target 62/group). For the path model, power for indirect effects estimated by Monte Carlo simulation (Schoemann et al., 2017): power ≈ 0.35 at $N = 79$; approximately 225 dyads required for 0.80 power.
Quantitative variables	11	Explain how quantitative variables were handled in the analyses. If applicable, describe which groupings were chosen and why	Section 2.6: all primary variables treated as continuous throughout. Normality assessed per group using Shapiro-Wilk test, skewness/kurtosis, and visual inspection; parametric vs. non-parametric tests selected accordingly. No continuous variables were categorized.
Statistical methods	12a	Describe all statistical methods, including those used to control for confounding	Section 2.6: group comparisons (Student's t-test / Welch's t-test / Mann-Whitney U / Fisher-Freeman-Halton); Spearman correlations and partial correlations; observed-variable path model estimated by FIML; Monte Carlo power simulation (Schoemann et al., 2017); HC3-robust OLS sensitivity analyses adjusting for child age, sex, and maternal education (Tables 4a/4b).
	12b	Describe any methods used to examine subgroups and interactions	No subgroup or interaction analyses were prespecified or conducted. Partial correlations controlling for child age, sex, and maternal education are reported in Tables 6–7 as a covariate-adjusted sensitivity check.
	12c	Explain how missing data were addressed	Section 2.6: missing data pattern described (19–23% on self-report instruments; 0% on clinician-rated WeeFIM and CBCL). Complete vs. incomplete cases compared (Table 9, Panel B). Path model estimated using FIML ($N = 79$); confirmed with multiple imputation

Item	No	Recommendation	Reported in manuscript / location
			(m = 20, Rubin's rules). Both approaches valid under missing-at-random assumption.
	12d	If applicable, describe analytical methods taking account of sampling strategy	Not applicable: consecutive sampling from a single center; no complex survey design or sampling weights used.
	12e	Describe any sensitivity analyses	Section 2.6 and Tables 4a/4b: HC3-robust OLS sensitivity analyses adjusting for child age, sex, and maternal education applied to all CBCL, PBI, PCRS, and CERQ outcomes. Multiple imputation (m = 20) used to confirm path model results alongside FIML estimation.
**Results**			
Participants	13a	Report numbers of individuals at each stage of study	Results, first paragraph: 141 child–mother dyads enrolled in total (79 CP group, 62 control group). Consecutive sampling; no loss to follow-up reported. Missing data confined to self-report instruments (Table 9, Panel A).
	13b	Give reasons for non-participation at each stage	Refusal rate was not tracked systematically. Participation was voluntary for all enrolled dyads.
	13c	Consider use of a flow diagram	No flow diagram included. Study flow described narratively in Section 2.3 and Results first paragraph.
Descriptive data	14a	Give characteristics of study participants and information on exposures and potential confounders	Table 1 (demographic characteristics of both groups with effect sizes), Table 2 (CP clinical profile, WeeFIM subscale scores, IFS scores), Table 3 (between-group scale score comparisons with effect sizes and 95% CIs).
	14b	Indicate number of participants with missing data for each variable of interest	Table 9, Panel A: missing data reported per instrument in the CP group (n available and % missing for each scale).
Outcome data	15*	Report numbers of outcome events or summary measures	Table 3: CBCL Total Problems and all syndrome/broadband subscales reported as median [min–max] or mean ± SD with between-group comparisons, effect sizes, and 95% CIs.
Main results	16a	Give unadjusted estimates and confounder-adjusted estimates and their precision (95% CI). Make clear which confounders were adjusted for and why they were included	Table 3: unadjusted comparisons with effect sizes [95% CI]. Tables 4a/4b: adjusted β [95% CI] controlling for child age, sex, and maternal education (selected based on observed group imbalances in Table 1). Table 8: standardized path coefficients and indirect effects with bootstrap 95% CIs.
	16b	Report category boundaries when continuous variables were categorized	Not applicable: no continuous variables were categorized in any analysis.
	16c	If relevant, consider translating estimates of relative risk into absolute risk for a meaningful time period	Not applicable: cross-sectional observational study; no risk ratios or hazard estimates calculated.

Item	No	Recommendation	Reported in manuscript / location
Other analyses	17	Report other analyses done — e.g. analyses of subgroups and interactions, and sensitivity analyses	Partial Spearman correlations adjusted for child age, sex, and maternal education (Tables 6–7); FIML path model confirmed by multiple imputation (m = 20); HC3-robust OLS sensitivity analyses (Tables 4a/4b). No subgroup or interaction analyses conducted.
**Discussion**			
Key results	18	Summarise key results with reference to study objectives	Section 4 (Discussion), first paragraph: key results summarized with explicit reference to primary aim (path model) and secondary aim (group comparisons). Non-significant indirect effects and single significant path (Self-Blame → IFS) highlighted.
Limitations	19	Discuss limitations of the study, taking into account sources of potential bias or imprecision. Discuss both direction and magnitude of any potential bias	Section 4.1 (Strengths, Limitations, and Future Directions): cross-sectional design, single-center and single-culture setting, sample size constraints (power ≈ 0.35 for indirect effects), common method variance, absence of GMFCS/MACS/CFCS, no data on maternal psychological support or school type.
Interpretation	20	Give a cautious overall interpretation of results considering objectives, limitations, multiplicity of analyses, results from similar studies, and other relevant evidence	Section 4 (Discussion) and Section 5 (Conclusions): findings interpreted as cross-sectional associations (not causal); null indirect effects interpreted as inconclusive given low power rather than evidence against mediation; self-blame framed as hypothesis-generating target for future longitudinal trials.
Generalisability	21	Discuss the generalisability (external validity) of the study results	Section 4.1: single-center, Turkey-specific sample limits generalizability; replication across diverse clinical and cultural contexts recommended in future studies.
**Other information**			
Funding	22	Give the source of funding and the role of the funders for the present study	Funding section: no external funding declared. Author contributions listed in Author Contributions section.
